# Baseline PSMA PET/CT parameters predict overall survival and treatment response in metastatic castration-resistant prostate cancer patients

**DOI:** 10.1007/s00330-025-11360-3

**Published:** 2025-01-22

**Authors:** Fleur Kleiburg, Lioe-Fee de Geus-Oei, Romy Spijkerman, Wyanne A. Noortman, Floris H. P. van Velden, Srirang Manohar, Frits Smit, Frank A. J. Toonen, Saskia A. C. Luelmo, Tom van der Hulle, Linda Heijmen

**Affiliations:** 1 Biomedical Photonic Imaging Group, University of Twente, Enschede, The Netherlands; 2 Department of Radiology, Section of Nuclear Medicine, Leiden University Medical Center, Leiden, The Netherlands; 3 Department of Radiation Science & Technology, Delft University of Technology, Delft, The Netherlands; 4 Multi-Modality Medical Imaging, University of Twente, Enschede, The Netherlands; 5 Department of Nuclear Medicine, Alrijne Hospital, Leiderdorp, The Netherlands; 6 Department of Oncology, Alrijne Hospital, Leiderdorp, The Netherlands; 7 Department of Medical Oncology, Leiden University Medical Center, Leiden, The Netherlands

**Keywords:** Prostatic neoplasms, Castration-resistant, PSMA-1007, Positron-emission tomography, Survival

## Abstract

**Objective:**

Metastatic castration-resistant prostate cancer (mCRPC) is a heterogeneous disease with varying survival outcomes. This study investigated whether baseline PSMA PET/CT parameters are associated with survival and treatment response.

**Methods:**

Sixty mCRPC patients underwent [^18^F]PSMA-1007 PET/CT before treatment with androgen receptor-targeted agents (ARTAs) or chemotherapy. Intensity-based parameters, volumetric parameters, metastatic sites and DmaxVox (distance between the two outermost voxels) from baseline PSMA PET/CT were collected, as well as age, Gleason score and laboratory parameters. Cox regression analysis evaluated their prognostic value for overall survival (OS). Additionally, a preliminary lesion-level analysis was done (*n* = 241 lesions) with lesion location and twelve radiomic features selected from previous literature. Logistic regression evaluated their association with PSMA PET/CT-based lesion progression after 3–4 months of treatment.

**Results:**

Total tumour volume (PSMA-TV) (HR = 1.41 per doubling [1.17–1.70]), total lesion uptake (TL-PSMA) (HR = 1.40 per doubling [1.16–1.69]) and DmaxVox (HR = 1.31 per 10 cm increase [1.07–1.62]) were prognostic for OS, each independent of baseline PSA level (HR = 0.82 per doubling [0.68–0.98]), haemoglobin level (HR = 0.68 per mmol/L increase [0.49–0.95]) and line of treatment. On lesion-level, location (prostate vs bone OR = 0.23 [0.06–0.83]) and SUV_mean_ (OR = 1.72 per doubling [1.08–2.75]) were independent prognostic markers for lesion progression, morphological and texture-based radiomic features were not.

**Conclusion:**

Baseline PSMA PET/CT scans have prognostic value in mCRPC patients and can potentially aid in treatment decision-making. DmaxVox can serve as a simpler alternative to PSMA-TV when automated segmentation software is not available. When combined with PSMA-TV, lower PSA levels indicated worse OS, which may be a marker of tumour dedifferentiation. Further research is needed to validate these models in larger patient cohorts.

**Key Points:**

***Question*** *mCRPC is a highly heterogeneous disease, requiring good prognostic markers*.

***Findings*** *PSMA-TV was the best independent prognostic marker for OS; maximum distance between lesions (DmaxVox) can be used as a simpler alternative*.

***Clinical relevance*** *Baseline PSMA PET/CT parameters representing tumour burden were independently associated with OS in mCRPC patients, providing prognostic insights for clinical decision-making. Although PSMA-TV was the best prognostic marker, DmaxVox can serve as an easier to obtain alternative*.

## Introduction

Metastatic castration-resistant prostate cancer (mCRPC) represents an advanced stage of prostate cancer, characterised by resistance to androgen deprivation therapy and the development of distant metastases [1]. Despite the availability of several novel therapies, all therapies are palliative and the prognosis remains poor with a median overall survival (OS) of 2–3 years [2]. The challenge in managing mCRPC patients is the heterogeneity of the clinical course, with some patients having indolent disease and long periods of disease control, while others have aggressive and rapidly progressing diseases with poor survival [2]. Therefore, the ability to accurately predict patient prognosis, including the likelihood of treatment response and survival, is important so that the expected treatment effects can be weighed against toxicity, patient burden, and associated costs for each individual patient. While several clinical parameters, such as Gleason score at initial biopsy [3] and prostate-specific antigen (PSA) [4], have been shown to have prognostic value in mCRPC patients, they do not capture the full heterogeneity of the disease.

A molecular imaging technique that may provide more accurate prognostic information is prostate-specific membrane antigen (PSMA) PET/CT, which is increasingly being used in clinical practice [1]. PSMA PET/CT imaging provides molecular characteristics of prostate cancer that reflect tumour biology [5, 6] and allows for the quantification and analysis of, for example, the intensity, distribution and heterogeneity of PSMA uptake. We hypothesised that disease characteristics on PSMA PET/CT can be used as an imaging biomarker to improve the prognostication of mCRPC patients and optimise treatment decision-making.

The primary objective of this study was to determine upfront the predictive and prognostic value of baseline PSMA PET/CT parameters in mCRPC patients, receiving either androgen receptor-targeted agents (ARTAs) or chemotherapy. Analysis was performed both at patient level and, as a preliminary study, at lesion level using a selection of hypothesis-driven parameters, including radiomic features.

## Methods

### Patient population

Patients with mCRPC treated with either an ARTA (enzalutamide or abiraterone) or chemotherapy (docetaxel or cabazitaxel) as first- or second-line treatment between 01-07-2019 and 31-06-2023 at the Leiden University Medical Center (Leiden, The Netherlands) and the Alrijne Hospital (Leiderdorp, The Netherlands) were included in this retrospective study. Administered treatment dosages were according to the European Association of Urology guidelines [1]. Clinical data such as age, medical history, pathology reports, radiology reports, laboratory results and survival data were retrieved from electronic patient records. All patients gave written informed consent for the use of their data for scientific research. The study protocol was approved on 03-03-2022 by the local institutional ethics committee.

### PSMA PET/CT imaging

In line with our local protocols, all patients underwent a [^18^F]PSMA-1007 PET/CT (in short: PSMA PET/CT) within 8 weeks before the start of treatment. PSMA PET/CT acquisitions and reconstructions were performed at the Alrijne Hospital using the 5-Ring Discovery MI PET/CT (GE Healthcare) in all included patients. According to the clinical scan protocol, injection-to-scan times were 60–120 min (depending on PSA, < 4 ng/mL: 120 min, 4–40 ng/mL: 80 min, > 40 ng/mL: 60 min) and injected doses of [^18^F]PSMA-1007 were 1.5–2.1 MBq/kg body weight (depending on BMI, < 25: 1.5 MBq/kg, 25–30: 1.8 MBq/kg, > 30: 2.1 MBq/kg [7]). First, a low-dose CT scan (15–550 mA, 120 kV) was performed from skull to mid-thigh for localisation and attenuation correction purposes, followed by a PET scan with 120 s per bed position. CT images were reconstructed at a 512 × 512 matrix with a slice thickness of 2.5 mm. PET images were reconstructed at a 256 × 256 matrix with a slice thickness of 2.78 mm. A Bayesian penalised-likelihood iterative image algorithm (Q.Clear with a beta value of 900) was used.

In line with our local protocols, all patients also received a PSMA PET/CT for imaging-based treatment response evaluation. In the case of ARTA treatment, PSMA PET/CT was performed after three months. In the case of chemotherapy, PSMA PET/CT was performed 4–6 weeks (absolute maximum: 8 weeks) after the last administered dose. When PSA progression (+25% and at least 2 ng/mL after at least three cycles [8]) or clinical deterioration (such as new-onset pain) was observed, a PSMA PET/CT was requested at that time.

### Image analysis and quantification

Volume of interest (VOI) delineation and feature extraction were done in baseline PSMA PET/CT scans using LIFEx software version 7.2 or higher [9]. Firstly, by applying a fixed absolute threshold of SUV = 4 and a minimum volume of 0.5 cm^3^, VOIs were automatically delineated [10]. Secondly, areas of physiological uptake were manually removed, using the low-dose CT as a reference. If liver lesions were present, they were manually delineated using the SUV_mean_ and standard deviation of a VOI of 3 cm in diameter in healthy liver tissue as a fixed threshold; SUV = 1.5 × SUV_mean,healthy liver_ + 2 × SD_healthy liver_ [11]. In each scan, the SUV_mean_, SUV_max_, PSMA-TV (total tumour volume), TL-PSMA (total lesion uptake: summed PSMA-TV × SUV_mean_) and DmaxVox (distance between the two lesions furthest apart using the two outermost voxels) were calculated. Here, DmaxVox represents tumour burden dissemination, a PSMA PET parameter that has been shown to be a prognostic factor in mCRPC patients treated with ^177^Lu-PSMA radioligand therapy [12]. Furthermore, the involvement of lymph nodes, bone and visceral tissues at sites of metastatic disease was noted [13, 14].

For the lesion-level analysis, only VOIs with at least 64 voxels were included to allow texture features to be included [15]. In each of the following locations, if present, the lesion with the highest SUV_max_ and highest PSMA-TV was selected: prostate, N1 lymph nodes, M1a lymph nodes, locoregional bones (pelvis and lumbar vertebrae), axial non-locoregional bones (cervical and thoracic vertebrae, claviculae, scapulae, ribs and base of the skull), appendicular bones (extremities and other parts of the skull), and visceral tissue. For each lesion, 12 PET parameters were extracted: 2 morphological (PSMA-TV, sphericity), 5 intensity-based (SUV_mean_, SUV_max_, SUV-kurtosis, SUV-IQR, and TL-PSMA) and 5 commonly used grey-level co-occurrence matrix (GLCM) texture features (energy, contrast, correlation, entropy, and homogeneity, from Haralick et al [16]). Specifically, sphericity, SUV-kurtosis, SUV-IQR, entropy and homogeneity were chosen, because these PSMA PET parameters have been associated with patient outcomes in mCRPC patients before [17–20]. Supplemental Table S1 describes each included PET parameter in more detail. Features were extracted using LIFEx software and were compliant with the Image Biomarker Standardization Initiative (IBSI) [21]. No feature selection was performed, as a limited number of features relative to the number of lesions [22] were selected from the literature. No voxel resampling was performed since the original voxel spacing was almost isotropic at 2.73 × 2.73 × 2.78 mm^3^. A fixed bin size of 0.5 g/mL was applied. To assess lesion response, PSMA-TV and TL-PSMA were also determined in each included lesion on the PSMA PET/CT acquired for treatment response evaluation.

### Clinical parameters

The previously described PSMA PET/CT parameters were compared with baseline PSA levels to assess their independent prognostic value. Age, Gleason score, baseline alkaline phosphatase (ALP) and haemoglobin (Hb) [3, 4] were also included in the analysis. In post-hoc analysis, PSA density was calculated for each patient by dividing baseline PSA level by PSMA-TV (ng/mL^2^) [23].

### Study endpoints

The primary endpoint of this study was OS, defined as the time from treatment initiation to time of death in months. Censored data used the time to the last hospital visit. At lesion level, the endpoint was PSMA PET/CT-based progression, which was defined as an increase of > 30% in lesion PSMA-TV or lesion TL-PSMA [24].

### Statistical analysis

All statistical analyses were performed in SPSS version 29 (IBM Corporation). For the summarising of retrieved data, descriptive statistics were used. Cox regression analysis and binary logistic regression analysis were performed to assess the prognostic value of parameters for OS and lesion progression. For input variables with skewness > 1, logarithmic (log_2_) transformation was used to transform skewed data into more normally distributed data required for statistical analyses [25]. A Spearman’s rho test was used to test the correlation between continuous variables, and a Chi-squared test was used to compare two categorical values. Kaplan-Meier curves and log-rank tests compared the difference in survival between groups. Statistical significance was reached when the *p*-value was < 0.05.

## Results

In total, 60 mCRPC patients were included in this study (Table 1). Thirty-one patients received ARTAs as either first-line (*n* = 27) or second-line treatment (*n* = 4); 29 patients received chemotherapy (median 6 cycles) as either first-line (*n* = 10) or second-line treatment (*n* = 19). The median follow-up time was 29 months (range 11–40 months). A total of 38 patients had died. Median OS was 21 months (range 4–31 months). No patients were lost to follow-up. For the baseline PSMA PET/CT scans, the median administered dose of [^18^F]PSMA-1007 was 153 MBq (IQR 127–173 MBq) and the median injection-to-scan time was 91 min (IQR 79–127 min).

**Table 1:** Patient characteristics (*n* = 60)

Characteristic	Value
Gleason score at diagnosis*	
≤ 7	14 (23%)
≥ 8	40 (67%)
Prior local treatment	
Radical prostatectomy	4 (7%)
Radiotherapy	24 (40%)
Prior treatment for mHSPC	
ADT	60 (100%)
Docetaxel	13 (22%)
Enzalutamide	6 (10%)
Abiraterone	1 (2%)
Time since diagnosis (years)	3.4 (2.1–5.9)
Age at start of treatment (years)	75 (68–78)
Baseline PSA level (ng/mL)	21.1 (8.1–55.6)
Baseline PSMA-TV (mL)	118 (41–328)
Sites of metastatic disease on baseline PSMA PET/CT scan	
Lymph node only	7 (12%)
Bone only	18 (30%)
Lymph node + bone	31 (52%)
Any visceral	4 (7%)
Current treatment	
ARTA	31 (52%)
Enzalutamide	25
Abiraterone	6
Chemotherapy	29 (48%)
Docetaxel	24
Cabazitaxel	5
Current line of treatment	
1st	37 (62%)
2nd	23 (38%)

Categorical data are presented as numbers (percentage), and continuous data as median (interquartile range)

*mHSPC* metastatic hormone-sensitive prostate cancer, *ADT* androgen deprivation therapy, *PSA* prostate-specific antigen, *PSMA-TV* total tumour volume on PSMA PET/CT, *ARTA* androgen receptor-targeted agent

* Gleason score unknown in six patients

### Prognostic baseline PSMA PET/CT markers for patient survival

The parameters SUV_mean_, PSMA-TV, TL-PSMA, PSA level and ALP level were log_2_ transformed. Univariate analysis for OS showed that PSMA-TV (HR = 1.395 per doubling), TL-PSMA (HR = 1.368 per doubling), DmaxVox (HR = 1.313 per 10 cm increase), metastatic sites (any visceral vs bone only; HR = 5.381), ALP level (HR = 1.531 per doubling), and Hb level (HR = 0.717 per unit increase) were prognostic markers (Table 2). SUV_mean_, SUV_max_, age, Gleason score, and PSA level had no prognostic value in this analysis. PSMA-TV was correlated with TL-PSMA (*r*_s_(58) = 0.952, *p* < 0.001) and DmaxVox (*r*_s_(58) = 0.701, *p* < 0.001), not with SUV_mean_ and SUV_max_. There was no difference in OS between patients treated with ARTAs and those treated with chemotherapy within the same line of treatment.

**Table 2:** Univariate Cox regression analyses OS with PSMA PET/CT and clinical parameters (*n* = 60 patients)

	OS		
Variable name (unit)	HR*	95% CI	*p*-value**
Baseline PSMA PET/CT parameters			
Log_2_ (SUV_mean_)	0.895	0.509–1.576	
SUV_max_	1.005	0.997–1.013	
Log_2_ (PSMA-TV (mL))	1.395	1.174–1.657	< 0.001
Log_2_ (TL-PSMA (SUV × mL))	1.368	1.149–1.629	< 0.002
DmaxVox (per 10 cm)	1.313	1.085–1.589	0.005
Sites of metastatic disease			0.024
Lymph node only	0.693	0.190–2.531	
Bone only	Ref	Ref	
Lymph node + bone	1.642	0.768–3.510	
Any visceral	5.381	1.624–17.83	0.006
Clinical parameters			
Age (years)	1.016	0.972–1.062	
Gleason score	1.178	0.842–1.648	
Log_2_ (baseline PSA (ng/mL))	1.108	0.959–1.281	
Log_2_ (baseline ALP (U/L))	1.531	1.093–2.146	0.013
Baseline Hb (mmol/L)	0.717	0.537–0.958	0.024

*Ref* reference group, *SUV* standardised uptake value, *PSMA-TV* total tumour volume, *TL-PSMA* total lesion uptake, *DmaxVox* distance between outermost voxels, *PSA* prostate-specific antigen, *ALP* alkaline phosphatase, *Hb* haemoglobin, *HR* hazard ratio

* HR, displayed per unit increase or, in case of log_2_ transformation, per doubling

** *p*-values are displayed when < 0.05

**Fig. 1:**
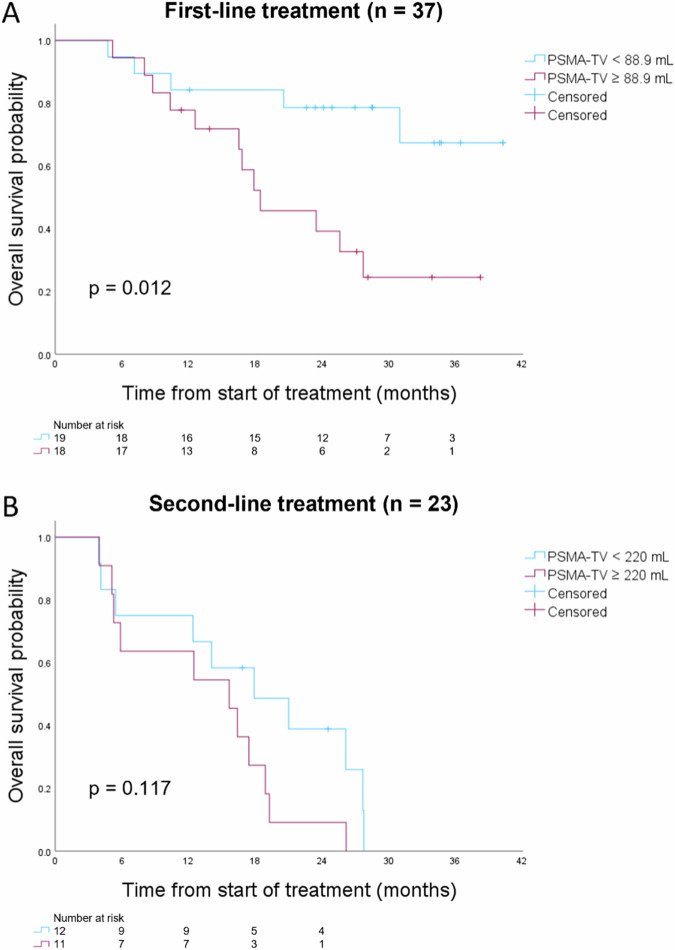
OS of patients receiving first-line (**A**) or second-line (**B**) treatment, subgrouped by median PSMA-TV

**Fig. 2:**
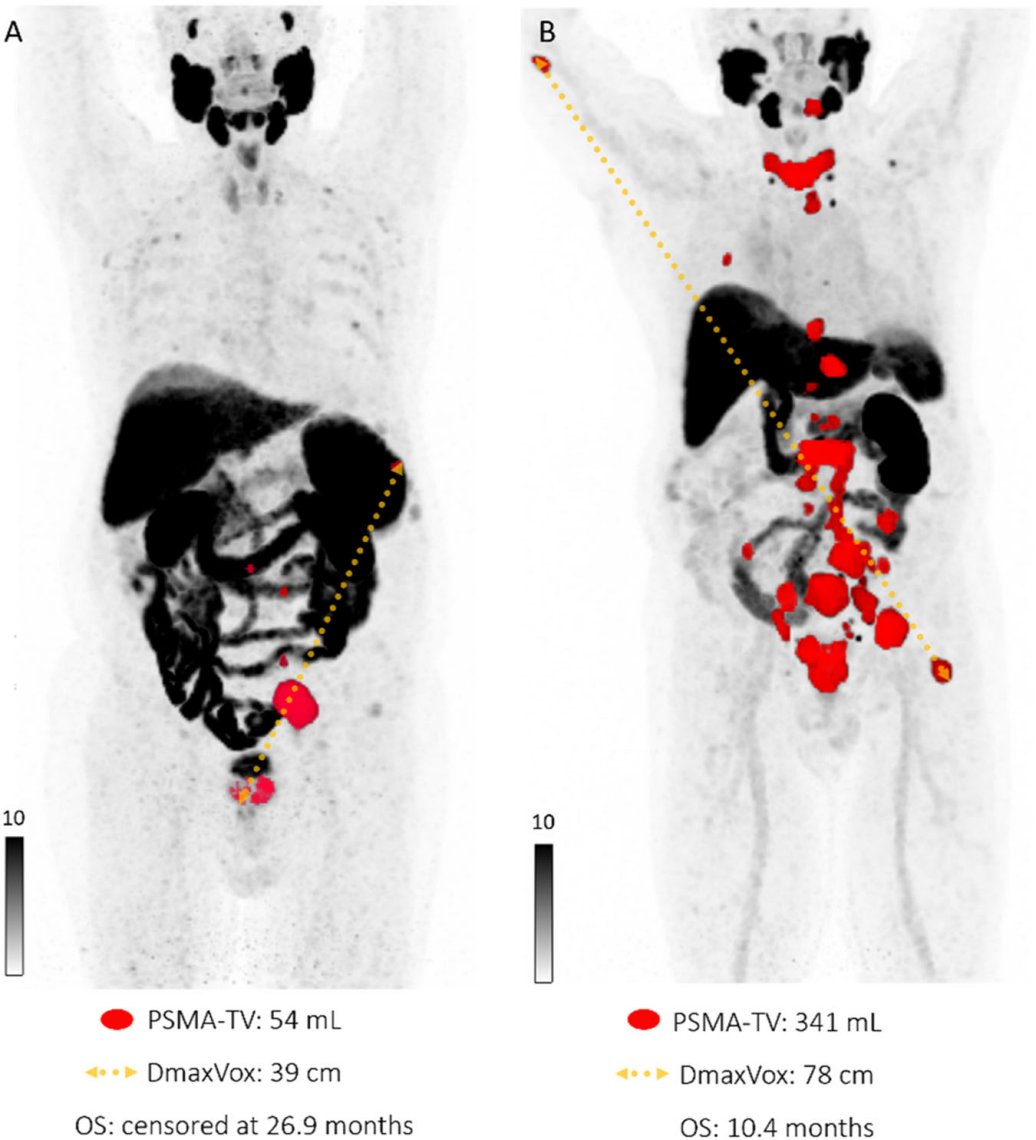
Maximum intensity projections (**A**, **B**) of baseline PSMA PET/CT in two different patients, both showing lymph node and bone metastases, before receiving enzalutamide as first-line mCRPC treatment. At diagnosis, both patients had Gleason 9 metastatic prostate cancer. Patient B, with a higher PSMA-TV (segmented in red) and DmaxVox (distance between outermost voxels, see dashed arrow) compared to patient A, had a lower OS

Multivariate Cox regression analysis for OS revealed that PSMA-TV (HR = 1.410 per doubling), PSA level (HR = 0.818 per doubling) and Hb level (HR = 0.680 per mmol/L increase) were all independent prognostic markers of OS, independent of line of treatment (Table 3). TL-PSMA (HR = 1.402 per doubling, *p* < 0.001) and DmaxVox (HR = 1.314 per 10 cm increase, *p* = 0.011) also remained significant prognostic markers of OS, independent of PSA level, Hb level and line of treatment (Supplemental Table S2). Multivariate models had lower −2 log-likelihood values than univariate models, indicating a better fit to the data. ALP levels lost significance in multivariate analysis with any other parameter. For interpretation and visualisation purposes, Table 4 shows the median OS of patients subgrouped by median PSMA-TV or median DmaxVox and Fig. 1 shows the survival distribution of patients subgrouped by median PSMA-TV. Figure 2 shows the PSMA PET/CT of two example patients.

**Table 3:** Multivariate Cox regression analysis for OS (*n* = 60 patients)

	OS		
Variable name (unit)	HR*	95% CI	*p*-value
Log_2_ (PSMA-TV (mL))	1.410	1.168–1.703	< 0.001
Log_2_ (baseline PSA (ng/mL))	0.818	0.683–0.978	0.028
Baseline Hb (mmol/L)	0.680	0.489–0.947	0.022
Line of treatment (second- vs first-line)	3.497	1.717–7.123	< 0.001

*PSMA-TV* total tumour volume, *PSA* prostate-specific antigen, *Hb* haemoglobin, *HR* hazard ratio

* HR displayed per unit increase or, in case of log_2_ transformation, per doubling

**Table 4:** Median OS subgrouped by median PSMA-TV or DmaxVox (distance between outermost voxels) in patients receiving first-line and second-line treatment

	First-line treatment (*n* = 37)		Second-line treatment (*n* = 23)	
	< Median	≥ Median	< Median	≥ Median
PSMA-TV range	4.1–88.9 mL	88.9–1095 mL	0.8–220 mL	220–3350 mL
Median OS	*	18.5 months	17.9 months	15.7 months
DmaxVox range	10.1–57.8 cm	57.8–86.1 cm	12.5–74.0 cm	74.0–96.7 cm
Median OS	*	23.5 months	17.9 months	12.5 months

* Not reached, median censoring time = 28.6 months

After obtaining these results, the PSA density (ng/mL^2^) was calculated for each patient. The median PSA density was 0.25 (IQR 0.10–0.67). Log_2_-transformation was done. In univariate analysis, PSA density was significantly associated with OS (HR = 0.803 per doubling, *p* = 0.006), and remained significant in multivariate analysis (HR = 0.760 per doubling, *p* < 0.001, Supplemental Table S2).

### Association of baseline PSMA PET/CT markers with lesion progression

For the lesion-level analysis, 241 lesions were selected; 113 lesions in patients receiving ARTAs and 128 lesions in patients receiving chemotherapy (Supplemental Table S3). Of 241 lesions, 76 showed progression on PSMA PET/CT; 3 of 32 prostate lesions (9%), 16 of 64 lymph node lesions (25%), 56 of 139 bone lesions (40%), and 1 of 6 visceral lesions (16%). There was no significant difference in the rate of progressive lesions between ARTA treatment (28%) and chemotherapy (34%, *p* = 0.313). Progression was seen in 22% of lesions receiving first-line treatment and 41% of lesions receiving second-line treatment. 57% of patients had a mixed response, with both responding and progressive lesions in the analysed dataset.

PSMA-TV, sphericity, SUV_mean_, SUV_max_, SUV-kurtosis, SUV-IQR, TL-PSMA, energy and contrast were log_2_ transformed. In logistic regression analysis with correction for line of treatment the lesion location, SUV_mean_, SUV_max_, SUV-IQR, energy, contrast, entropy and homogeneity were significantly associated with lesion progression, while PSMA-TV, sphericity, SUV-kurtosis, TL-PSMA and correlation were not (Supplemental Table S4). Specific lymph node location (N1 vs M1a) and bone location (locoregional vs axial non-locoregional vs appendicular) were also not associated with lesion progression. The PET parameters SUV_mean_, SUV_max_, SUV-IQR, energy, contrast, entropy and homogeneity were all highly correlated (all *p* < 0.003).

A multivariate logistic regression analysis demonstrated that lesion location (bone vs prostate: OR = 0.23), SUV_mean_ (OR = 1.72 per doubling) and line of treatment (second- vs first-line: OR = 2.16) were independently associated with lesion progression (Table 5).

**Table 5:** Multivariate logistic regression analysis for imaging-based lesion progression after 3–4 months of treatment

	Lesion progression (*n* = 241 lesions)		
Variable name (unit)	OR*	95% CI	*p*-value^**^
Location			0.039
Prostate	0.231	0.064–0.825	0.024
Lymph node	0.526	0.265–1.044	
Bone	Ref	Ref	
Visceral	0.277	0.030–2.519	
Log_2_ (SUV_mean_)	1.724	1.080–2.753	0.022
Line of treatment (second- vs first-line)	2.158	1.196–3.892	0.011

*Ref* reference group, *SUV* standardised uptake value, *OR* odds ratio

* OR displayed per unit increase or, in case of log_2_ transformation, per doubling

^**^ *p*-values are displayed when < 0.05

## Discussion

The results of this study showed that baseline PSMA PET/CT scans provide prognostic information for mCRPC patients treated with ARTA or chemotherapy. For each patient, it is important to weigh the expected treatment benefit against the risk of toxicity and associated costs. Prognostic factors currently considered before initiating a new line of treatment include the presence of disease-related symptoms, PSA levels, PSA doubling time and the presence of visceral metastases [1]. However, response to therapy and survival rates among mCRPC patients remain heterogeneous [2] and more accurate predictive and prognostic biomarkers are needed. This study showed that higher PSMA-TV (HR = 1.41 per doubling) was associated with worse OS, independent of line of treatment and PSA level. The same was true for TL-PSMA and DmaxVox, also markers of disease burden. The significant association between TL-PSMA and survival was mainly determined by PSMA-TV, as SUV_mean_ was not associated with survival. Combining PET parameters with the clinical parameters PSA and Hb level in multivariate analysis resulted in improved prognostic models. Interestingly, while PSA levels had no prognostic value in univariate analysis, lower PSA levels (HR = 0.8 per doubling) were associated with worse OS in multivariate analysis when combined with PSMA PET parameters representing disease extensiveness (e.g. PSMA-TV). This was confirmed after calculating PSA density (PSA/PSMA-TV), which was also associated with worse OS and may represent a marker of tumour dedifferentiation. Aggarwal et al also observed dedifferentiation in low PSA-secreting mCRPC and an association with shorter OS [26].

The prognostic value of PSMA PET/CT parameters, particularly PSMA-TV, in mCRPC patients receiving ARTA treatment or chemotherapy, has also been recognised in previously published literature. In two studies involving 54 mCRPC patients receiving first-line docetaxel or ARTA treatment [27] and 32 mCRPC patients receiving second-line cabazitaxel treatment [28], PSMA-TV was the only independent prognostic marker for OS. In contrast, baseline PSA level, Gleason score, and ECOG performance status had no prognostic value [27]. Has Simsek et al found that PSMA-TV and age were independent prognostic factors of OS in 52 mCRPC patients receiving first-line docetaxel treatment, while PSA level was not [29]. The association between lower Hb levels and worse OS has also been observed previously in mCRPC patients receiving first-line chemotherapy [4]. Until now, the prognostic value of DmaxVox, a dissemination feature representing the metastatic spread of prostate cancer cells, has only been investigated in mCRPC patients receiving ^177^Lu-PSMA radioligand therapy [12]. In our study, we showed that DmaxVox was also a prognostic marker in mCRPC patients receiving ARTA treatment or chemotherapy, with higher DmaxVox being associated with worse OS. Interestingly, DmaxVox had a similar prognostic value compared to PSMA-TV. Especially since tumour segmentations to obtain volumetric PET parameters can be time-consuming and labour-intensive, DmaxVox may be an easy-to-implement prognostic biomarker in clinical centers where automated segmentation software is not (yet) available.

By performing a preliminary analysis using location and radiomic features at lesion level, we aimed to improve our understanding of CRPC lesion characteristics associated with progression, which in turn could potentially improve our understanding patient level. This study found that lesion location was associated with lesion progression on PSMA PET/CT after 3–4 months, with bone lesions having the highest progression rate. We hypothesise that this is caused by the relatively high density and low vascularisation of bone and by the bone marrow microenvironment, which may protect prostate cancer cells from treatment effects [30]. As for visceral lesions, the included number of lesions (*n* = 6) was too small to draw any conclusions. Although several GLCM features representing tumour heterogeneity were associated with lesion progression in univariate analysis, they lost significance in multivariate analysis. Furthermore, lesion volume was not associated with progression, in contrast to PSMA-TV at patient level. As this was a radiomic analysis using twelve selected features from the literature, the potential of a radiomic analysis testing a wide variety of image features was not explored. We suggest that larger numbers of lesions and more advanced methods (e.g. machine learning or deep learning models) are needed to draw robust conclusions and to overcome the challenge of multicollinearity between PET parameters. The use of image filters can also potentially improve textural signal-to-noise ratios and may be considered for future research. Hopefully, improving our understanding of predictive markers for lesion progression, it can be helpful for treatment decision-making, prognostication and therapy response monitoring in the future.

To the best of our knowledge, this is the first study to investigate the prognostic value of baseline PSMA PET/CT scans using a fluorine-18 tracer. Although PET parameters are expected to remain prognostic markers when different tracers are used, the SUV values can differ between PSMA tracers and image reconstruction methods, and volumes can change depending on the segmentation methods. It is important to note that hazard ratios and odds ratios may be affected by these differences. Harmonisation of PET images and parameters, e.g. using image reconstructions compliant with EARL [31], and reaching consensus on the optimal segmentation method will remain key focus points.

Limitations of this study include the retrospective nature, variability in injection-to-scan times, and heterogeneity in terms of prior systemic lines of therapy and administered therapies. However, results were adjusted for prior lines of therapy and no differences were seen between patients treated with ARTA and those treated with chemotherapy. Therefore, we showed that PSMA PET/CT can provide robust prognostic parameters across multiple clinical mCRPC settings. For lesion-level analysis, limitations include the lack of validation data and the inclusion of a specific selection of lesions. The specific selection of lesions was done because texture features could not be extracted from lesions < 64 voxels [15], which with the voxel size in this study corresponds with a lesion volume of 11 × 11 × 11 mm. With the development of higher-resolution PET cameras and advanced image reconstruction algorithms, the extraction of texture features from smaller lesions may be possible in the future. The specific selection of lesions was also done for practical reasons, as many PSMA PET/CT scans contained multiple lesions merged into a single VOI, and it would be too time-consuming to split all lesions for separate analysis. As more automated segmentation tools become available, the segmentation of all individual lesions will become easier in the future as well.

## Conclusion

In this study of 60 mCRPC patients who received a baseline PSMA PET/CT before treatment with ARTAs or chemotherapy, several prognostic factors associated with worse OS were identified, independent of the line of treatment. These factors include higher baseline PSMA-TV, TL-PSMA and DmaxVox, in combination with lower PSA and Hb levels. DmaxVox can be used as an easier alternative to PSMA-TV when automated segmentation software is not available. In combination with PSMA-TV, lower PSA levels indicated a worse OS, which may be a marker of tumour dedifferentiation. The results of this study can be used as input for the development of a prediction tool for mCRPC patients.

## Supplementary information

ELECTRONIC SUPPLEMENTARY MATERIAL

## Abbreviations

ARTAs: Androgen receptor-targeted agents
mCRPC: Metastatic castration-resistant prostate cancer
OS: Overall survival
PSA: Prostate-specific antigen
PSMA: Prostate-specific membrane antigen
TL-PSMA: Total lesion uptake
PSMA-TV: Total tumour volume
VOI: Volume of interest
