# Supplementary material for: Baseline PSMA PET/CT parameters predict overall survival and treatment response in metastatic castration-resistant prostate cancer patients
Source: Eur Radiol. 2025 Jan 22;35(7):4223–32. doi: 10.1007/s00330-025-11360-3 (PMC12165979; doi:10.1007/s00330-025-11360-3)
Supplement: Supplementary file 1 — ELECTRONIC SUPPLEMENTARY MATERIAL [file 330_2025_11360_MOESM1_ESM.pdf]

Baseline PSMA PET/CT parameters predict overall survival and treatment response in metastatic castration-resistant prostate cancer patients

ELECTRONIC SUPPLEMENTARY MATERIAL

Table S1

For patient-level analysis	
**Parameter**	**Explanation (IBSI code [20])**
**Morphological**	
PSMA-TV	Total tumour volume in mL (RNU0)
DmaxVox	The distance in cm between the two lesions that are the furthest apart, using the outermost voxels (no IBSI code)
**Intensity-based***	
SUV_{mean}	The mean grey level intensity within the total tumour volume (Q4LE)
SUV_{max}	The maximum grey level intensity within the total tumour volume (84IY)
TL-PSMA	The product of SUV_{mean} and total tumour volume in mL (no IBSI code)
For lesion-level analysis	
**Parameter**	**Explanation (IBSI code [20])**
**Morphological**	
PSMA-TV	Lesion volume in mL (RNU0)
Sphericity	A measure of the roundness of the shape of the tumour region relative to a sphere; higher values correspond to higher roundness (QCFX)
**Intensity-based***	
SUV_{mean}	The mean grey level intensity within the VOI (Q4LE)
SUV_{max}	The maximum grey level intensity within the VOI (84IY)
Kurtosis	A measure of peakedness of the grey level distribution; higher values mean that the mass of the distribution is concentrated towards the tail(s), lower values mean that the mass of the distribution is concentrated towards a spike near the mean value (IPH6)
Interquartile range	The difference between the 25th and 75th percentiles of the grey level distribution (SALO)
TL-PSMA	The product of SUV_{mean} and volume in mL (no IBSI code)

GLCM**	
Energy	Or Angular Second Moment; a measure of homogeneous patterns in the VOI, a greater energy implies that there is a higher frequency of neighbouring intensity value pairs (8ZQL)
Contrast	A measure of the local intensity variation; larger values correlate with a higher differences in grey level intensity values among neighbouring voxels (ACUI)
Correlation	The linear dependency of grey level values to their respective voxels in the GLCM; higher values mean higher correlation (NI2N)
Entropy	A measure of randomness in neighbourhood grey level intensity values (TU9B)
Homogeneity	Or Inverse Difference; a measure of homogeneity of grey level intensity values, higher values mean more uniform grey levels (IB1Z)

*The intensity of a pixel or voxel is also called a grey level.
** The GLCM is calculated from 13 different directions in 3D and the GLCM features are average values over these 13 directions.
PSMA-TV: PSMA-based tumour volume, SUV: standardised uptake value, VOI: volume of interest, TL-PSMA: PSMA-based total lesion uptake. GLCM: grey level co-occurrence matrix.

Table S2

Multivariate Cox regression analyses for overall survival with PSMA PET/CT and clinical parameters.
* HR: hazard ratio, displayed per unit increase or, in case of log_2 transformation, per doubling. ** p-values are displayed when < 0.05.

	OS		
**Variable name (unit)**	**HR***	**95% C.I.**	**p-value****
Log_2(TL-PSMA (SUV×mL))	1.402	1.161-1.693	< 0.001
Log_2(baseline PSA (ng/mL))	0.826	0.693-0.985	0.034
Baseline Hb (mmol/L)	0.647	0.464-0.903	0.010
Line of treatment (second- versus first-line)	3.406	1.679-6.909	< 0.001
DmaxVox (per 10 cm)	1.314	1.065-1.619	0.011
Log_2(baseline PSA (ng/mL))	0.864	0.724-1.030	
Baseline Hb (mmol/L)	0.695	0.496-0.975	0.035
Line of treatment (second- versus first-line)	3.180	1.521-6.645	0.002
Log_2(PSA density (ng/mL^2))	0.760	0.652-0.885	<0.001
Baseline Hb (mmol/L)	0.640	0.465-0.883	0.006
Line of treatment (second- versus first-line)	3.941	1.984-7.828	< 0.001

Table S3

Lesion characteristics (n = 241). Categorical data are presented as number (percentage), continuous data as median (interquartile range).

**Characteristic**	**Value**	
Received treatment		
ARTA	113	(47%)
Chemotherapy	128	(53%)
Line of treatment		
1st	122	(51%)
2nd	119	(49%)
Lesion location		
Prostate	32	(13%)
Lymph node	64	(27%)
N1	*21*	
M1a	*43*	
Bone	139	(58%)
Locoregional	*55*	
Axial, non-locoregional	*59*	
Appendicular	*25*	
Visceral	6	(3%)
SUV_{mean}	9.4	(7.3 – 13.6)
SUV_{max}	30.0	(16.3 – 47.5)
PSMA-TV (mL)	7.7	(3.4 – 21.9)
TL-PSMA (SUV×mL)	76.4	(30.4 – 239.4)

Table S4

Univariate logistic regression analyses for imaging-based lesion progression after 3-4 months of treatment, all corrected for line of treatment. * OR: odds ratio, displayed per unit increase or, in case of log_2 transformation, per doubling. ** p-values are displayed when < 0.05. Ref = reference group.

	Lesion progression (n = 241 lesions)		
**Variable name (unit)**	**OR***	**95% CI**	**p-value****
**Location**			0.013
Prostate	0.169	0.049-0.589	0.005
Lymph node	0.553	0.282-1.086	
Bone	ref	ref	
Visceral	0.232	0.026-2.076	
**Morphological**			
Log_2(PSMA-TV (mL))	0.977	0.835-1.142	
Log_2(Sphericity)	1.162	0.472-2.859	
**Intensity-based**			
Log_2(SUV_{mean})	1.973	1.265 -3.079	0.003
Log_2(SUV_{max})	1.400	1.049-1.868	0.022
Log_2(SUV-kurtosis)	0.808	0.614-1.064	
Log_2(SUV-IQR)	1.352	1.071 -1.707	0.011
Log_2(TL-PSMA (SUV×mL))	1.056	0.915-1.219	
**GLCM**			
Log_2(Energy)	0.837	0.713-0.982	0.029
Log_2(Contrast)	1.168	1.035-1.318	0.012
Correlation	0.812	0.150-4.403	
Entropy	1.181	1.004 -1.388	0.044
Homogeneity	0.065	0.005-0.802	0.033
